# Effectiveness of Butorphanol in alleviating intra- and post-operative visceral pain following microwave ablation for hepatic tumor: a dual-central, randomized, controlled trial

**DOI:** 10.1038/s41598-024-56876-8

**Published:** 2024-03-19

**Authors:** Bibo Wang, Neng Wang, Zhiyue Zhao, Shengxi Huang, Qiang Shen, Sheng Liu, Pingsheng Zhou, Lu Lu, Guojun Qian

**Affiliations:** 1 Department of Minimally Intervention Therapy, Eastern Hepatobiliary Surgery Hospital, Second Military Medical University/NAVAL Medical University, Shanghai, China; 2 Department of Medical Oncology, Jinling Hospital, Affiliated Hospital of Medicine School, Nanjing University, Nanjing, China; 3 Department of Special Clinic, Affiliated Hospital of Medicine School, Jinling Hospital, Nanjing University, Nanjing, China

**Keywords:** Butorphanol, Microwave ablation, Hepatic tumor, RCT, Cancer, Gastrointestinal diseases, Hepatology

## Abstract

Many patients who underwent hepatic percutaneous microwave ablation (MWA) reported experiencing pain during the procedure. This study utilized a well-designed multicentral, randomized, and placebo-controlled format to investigate the effects of Butorphanol. Patients who underwent MWA were randomly assigned to either Butorphanol or normal saline group. The primary outcomes of the study were assessed by measuring the patients' intraoperative pain levels using a 10-point visual analog scale (VAS). Secondary outcomes included measuring postoperative pain levels at the 6-h mark (VAS) and evaluating comprehensive pain assessment outcomes. A total of 300 patients were divided between the control group (n = 100) and the experimental group (n = 200). Butorphanol showed statistically significant reductions in intraoperative pain levels compared to the placebo during surgery (5.00 ± 1.46 vs. 3.54 ± 1.67, P < 0.001). Significant differences were observed in postoperative pain levels at the 6-h mark and in the overall assessment of pain (1.39 + 1.21 vs. 0.65 + 0.81, P < 0.001). Butorphanol had a significant impact on reducing the heart rate of patients. The empirical evidence supports the effectiveness of Butorphanol in reducing the occurrence of visceral postoperative pain in patients undergoing microwave ablation for hepatic tumor. Furthermore, the study found no noticeable impact on circulatory and respiratory dynamics.

## Introduction

Hepatocellular carcinoma (HCC) stands as a formidable global public health predicament, positioning itself as the second most prevalent etiology of cancer-related mortality^1^. Locoregional interventional therapies, defined as imaging-guided liver-tumour-directed procedures, play a leading part in the management of HCC, and it is estimated that 50–60% of patients with HCC might receive those treatments in their lifespan, globally^2^. Minimally-invasive approaches, such as transarterial chemoembolization (TACE), transarterial embolization (TAE), transarterial radioembolization (TARE), and ablation may be indicated based on patient clinical status and tumor characteristics^3^. In general, locoregional liver-directed treatments provide less morbidity than traditional surgical options while also improving outcomes compared to traditional systemic therapies^4^. Subsumed within the landscape of local ablative options, techniques such as radiofrequency ablation (RFA) and microwave ablation (MWA) hold sway in the management of early-stage hepatic malignancies^5^. MWA, characterized by its rapid temperature elevation, augmented peak thermal levels, and expansive ablation zones relative to RFA, confers an advantageous capacity for managing larger tumor dimensions. Nevertheless, the postsurgical trajectory following MWA, particularly concerning pain experiences subsequent to central lesion ablations^6^, engenders disparities in pain severity and persistence. These observations underscore the exigency of preemptive strategies to address postoperative pain and its attendant issues of unexpected discomfort or insufficient sedation, despite judicious perioperative care.

Butorphanol is a mixed opioid receptor agonist and antagonist that acts on κ receptors^7^.The pharmacological attributes of Butorphanol, characterized by its mild impact on cardiopulmonary dynamics and demonstrated efficacy in attenuating mechanical traction-induced discomfort, culminate in an ameliorative effect on postoperative nausea and vomiting rates^8^. A trans-nasal dosage form of Butorphanol (Stadol nasal spray) was developed to avoid hepatic first pass metabolism, achieve rapid absorption, increase systemic bioavailability, and provide a convenient mode of administration. Butorphanol is safe and even in patients with liver dysfunction, the initial dose of Butorphanol nasal spray may not need to be adjusted^9^. Importantly, it substantively attenuates visceral postoperative pain. However, sedation can also cause dizziness, drowsiness, and other adverse reactions during recovery^10^.

In the context of this meticulously designed randomized controlled trial, the objective is to corroborate the substantive improvements afforded by Butorphanol in the domain of postoperative visceral pain relief, thereby concomitantly reducing incidence rates and ameliorating pain intensities. This, in turn, engenders a salutary influence on the overall surgical experience.

## Results

### Demographic and perioperative characteristics of patients

**Figure 1:**
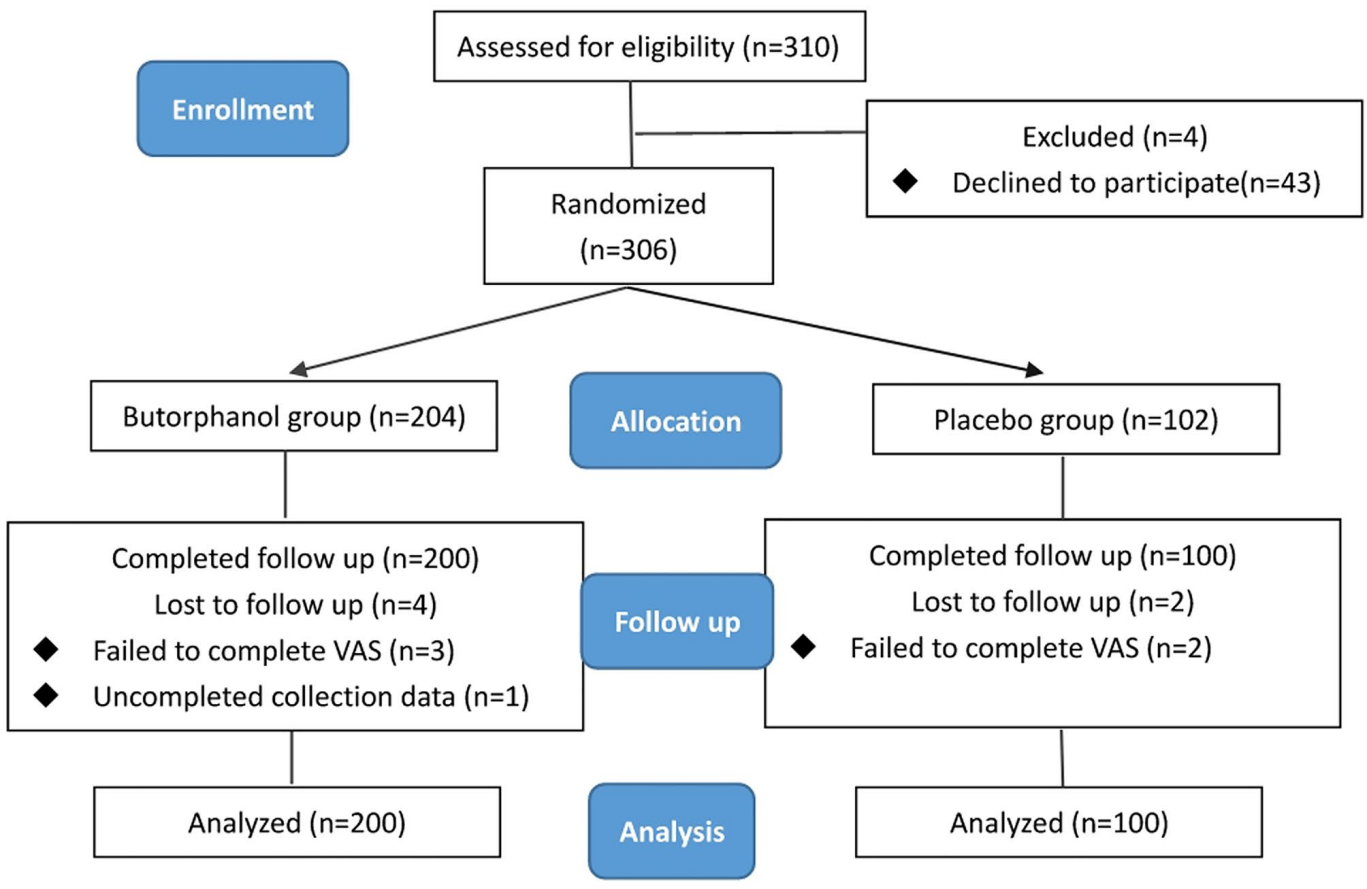
CONSORT flow diagram. CONSORT: Consolidated Standards of Reporting Trials.

Demographic and perioperative characteristics of patients were compiled from an ITT population consisting of 310 patients consecutively registered at two hospitals between 2022 and 2023. Ultimately, all 306 patients were successfully randomized, with 102 assigned to the control group and 204 to the experimental group. Four patients of the experimental group and 2 patients of the control group were lost to follow-up during the study, ensuring the inclusion of data from all participants in the analysis. A total of 300 patients in the two groups completed the study (Fig. 1). Two groups exhibited comparable demographic features, baseline assessments, and intraoperative details (Table 1).

**Table 1:** Comparison of baseline clinical characteristics between the two groups.

Characteristics	Control group n = 100	Experimental group n = 200	p-value
Gender, n (%)			0.49
Male	83(83.00)	172(86.00)	
Female	17(17.00)	28(14.00)	
Age (years)	60.34 ± 11.05	61.74 ± 10.67	0.29
Height(cm)	168.26 + 6.45	169.72 + 7.42	0.096
Weight(kg)	68.68 + 11.69	70.1 + 10.87	0.30
BMI	24.16 + 3.23	24.31 + 3.28	0.71
Smoking history, n (%)	29(29.00)	46(23.00)	0.26
Drinking history, n (%)	26(26.00)	54(27.00)	0.85
Hypertension, n (%)	30(30.00)	51(25.50)	0.41
Diabetes mellitus, n (%)	21(21.00)	51(25.50)	0.39
Diagnosis, n (%)			0.22
PLC	80(80.00)	174(87.00)	
MLC	17(17.00)	20(10.00)	
Benign tumors	3(3.00)	6(3.00)	
Diameter of the largest lesion (mm)	22.79 + 11.69	23.35 + 11.59	0.69
Number of tumors	1.38 + 0.63	1.44 + 0.73	0.52

BMI, body mass index; PLC, primary liver cancer; MLC, metastatic liver cancer; Chi-square test and Fisher’s exact test were used for comparisons between groups. Student’s t-test was used for analysis when the data were in normal distribution.

### Visceral pain

Primarily, a noteworthy disparity emerged between the two groups in terms of the requirement for additional analgesics during both the intraoperative and postoperative periods. Specifically, 19 patients in the control group necessitated supplementary analgesia due to intolerable intraoperative pain, contrasting with merely 8 patients in the experimental group. This discernible discrepancy is illustrated in Table 2 and attains statistical significance (P < 0.0001).

**Table 2:** Comparison of treatment parameters and Butorphanol usage between groups.

Characteristics	Control Group n = 100	Experimental Group n = 200	*p*-value
Ablation duration, (minutes) mean ± SD	17.38 + 6.36	18.63 + 8.21	0.18
Ablation probes			0.38
60 W	99 (99.00)	195 (97.50)	
70 W	1 (1.00)	5 (2.50)	
Dose level of butorphanol (mg), mean ± SD		4.00 ± 0	
Number of patients needed additional painkiller during surgery	19 (19.00)	8 (4.00)	** < 0.0001**
Number of patients needed additional painkiller after surgery	13 (13.00)	8 (4.00)	** < 0.0001**

Chi-square test and Fisher’s exact test were used for comparisons between groups. Student’s t-test was used for analysis when the data were in normal distribution. Significant values are in bold.

Subsequently, a substantial distinction was observed in the intraoperative pain ratings between the two groups (mean pain levels: control group 5.00 ± 1.46, experimental group 3.54 ± 1.67, p < 0.001, Table 3). The nasal administration of Butorphanol resulted in subjectively alleviated patient pain levels. The divergence persisted at the 6-h postoperative mark. Notably, a remarkable proportion of patients (106 individuals) receiving Butorphanol reported complete relief from pain, surpassing the control group's count of 18 patients. The divergence in postoperative pain relief maintained its statistical significance (p < 0.001).

**Table 3:** Comparison of efficacy outcomes of two groups.

	Control Group n = 100	Experimental Group n = 200	p-value
Pain level before surgery	0.01 + 0.1	0	0.16
Primary outcome			
Pain level during surgery	5.00 + 1.46	3.54 + 1.67	< 0.001
No pain (0)	0 (0.00)	0 (0.00)	< 0.001
mild pain (1–3)	17 (17.00)	105 (52.50)	
moderate pain (4–6)	68 (68.00)	83 (41.50)	
severe pain (7–10)	15(15.00)	12 (6.00)	
ORR(95%CI)	93 + 2.60	100	
DCF(95%CI)	99 + 1.00	100	
Secondary outcome			
Pain level after surgery 6 h	1.39 + 1.21	0.65 + 0.81	< 0.001
Pain evaluation			
Complete relief	18	106	< 0.001
Partial relief	75	194	
Stable disease	6	0	
Progressive disease	1	0	

Chi-square test and Fisher’s exact test were used for comparisons between groups. Student’s t-test was used for analysis when the data were in normal distribution.

### Biochemical indexes

Comparative assessment of biochemical indices (TBil, DBil, ALT, AST, Albumin, Creatinine, Glucose) at preoperative and postoperative time points yielded no conspicuous differences between the two groups. These results underscore that the intranasal administration of 4 mg Butorphanol did not exert a significant influence on patients' physiological functions (Table 4).

**Table 4:** Comparison of biochemical indexes of two groups.

	Time points	Control group n = 100	Experimental group n = 200	p-value
TBil	Before-surgery	16.83 + 7.86	15.81 + 7.80	0.29
	Post-surgery	30.02 + 13.02	28.43 + 11.58	0.29
DBil	Before-surgery	5.42 + 2.83	5.17 + 2.88	0.47
	Post-surgery	9.62 + 4.51	9.33 + 4.17	0.58
ALT	Before-surgery	29.06 + 21.29	25.77 + 17.03	0.15
	Post-surgery	147.14 + 125.95	159.82 + 147.54	0.47
AST	Before-surgery	29.61 + 17.21	26.51 + 12.02	0.070
	Post-surgery	188.36 + 158.85	194.98 + 176.24	0.75
Albumin	Before-surgery	41.50 + 4.01	41.96 + 4.67	0.40
	Post-surgery	39.66 + 4.21	39.87 + 4.22	0.69
Creatinine	Before-surgery	73.72 + 18.39	74.4 + 19.57	0.77
	Post-surgery	71.58 + 17.94	74.19 + 19.66	0.27
Glucose	Before-surgery	6.27 + 2.60	6.71 + 2.75	0.18
	Post-surgery	6.02 + 1.81	6.22 + 2.09	0.42

Student’s t-test was used for analysis when the data were in normal distribution.

### ***Perioperative monitoring parameters (MAP, SpO***_***2***_***, HR, RR)***

**Figure 2:**
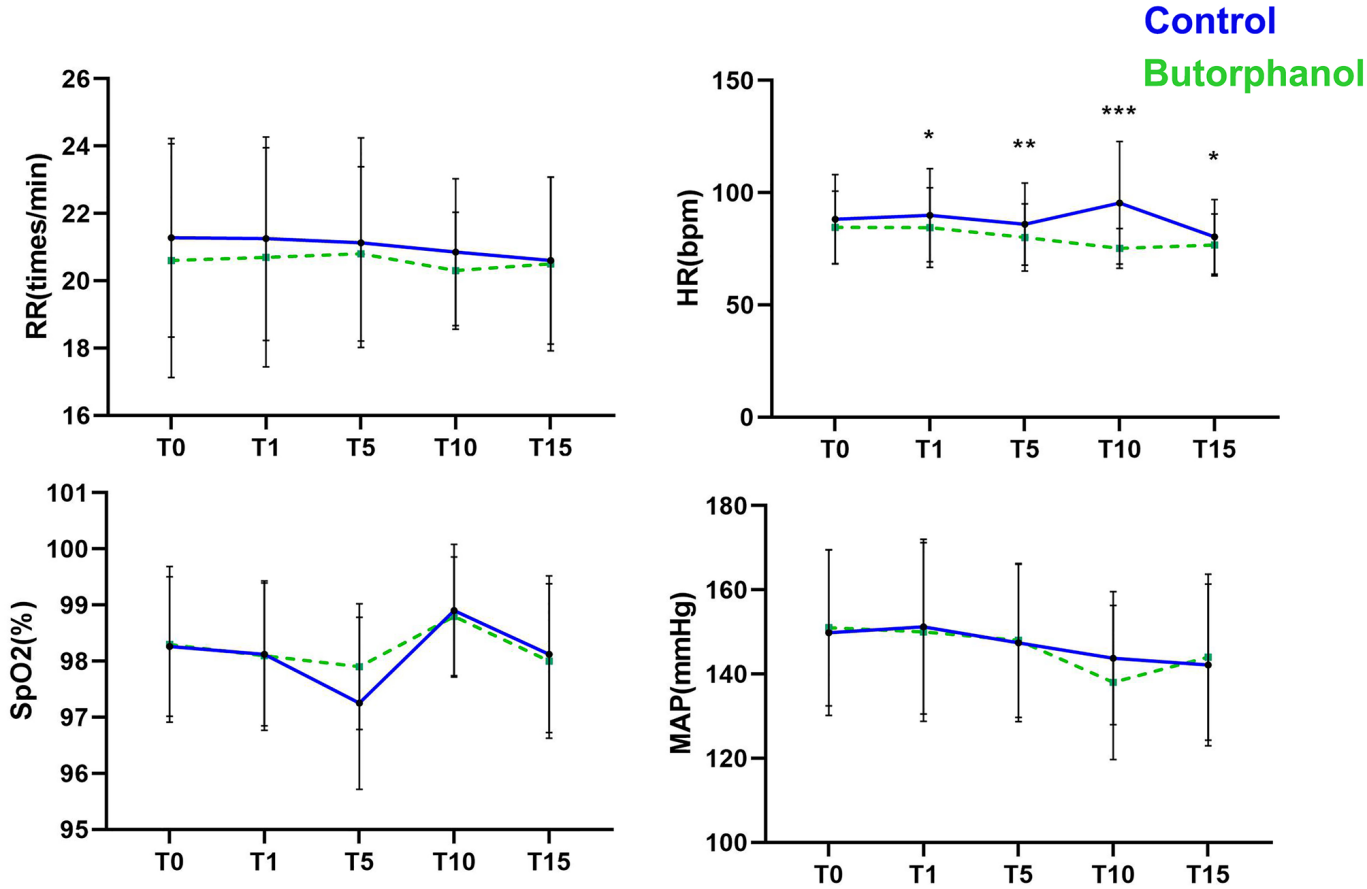
Changes in MAP, HR, SpO_2_, and RR. MAP, HR: Normal distribution, mean, and SD. SpO_2_, RR: Nonnormal distribution, median, and upper/lower limit. MAP, mean arterial pressure; HR, heart rate; RR, respiratory rate; SpO_2_, pulse oxygen saturation. T0, at admission; T1, beginning of microwave ablation; T5, 5 min after microwave ablation; T10, 10 min after microwave ablation; T15, 15 min after microwave ablation. (*P < 0.05, **P < 0.01, ***P < 0.001).

The MAP, SpO_2_, and RR were not significantly diferent between the two groups of patients at the five time points. Only after beginning (T1, P = 0.036), 5 min (T5, P = 0.0071), 10 min (T10, P < 0.001) of Butorphanol administration and at the end of the operation (T15, P = 0.025) was the HR of experiment group slightly lower than that of control group (Fig. 2).

### Adverse events

Only one patient in the Butorphanol group developed fatigue during the operation. After 24 h of recovery in the Butorphanol group, the number of people with nausea or vomiting, abdominal bloating, dizziness, or headache was 2, 7, 3, and 5, respectively. In control group, the number of people with nausea or vomiting, abdominal bloating, dizziness, or headache was 0, 4, 2, and 3. No cases of hypoxemia or body movements were reported. There were no signifcant diferences between the two groups in adverse events (χ2 = 6.345, P = 0.282).

## Discussion

This study indicates that Butorphanol reduces the incidence and severity of pain during and after percutaneous liver microwave ablation procedures, with no significant impact on patients' circulatory and respiratory functions. Intranasal administration of Butorphanol neither affects biochemical markers nor increases adverse reactions.

Microwave ablation is a minimally invasive procedure where microwave energy is used to heat and destroy abnormal tissues or tumors^11^. It has gained popularity due to its efficacy, minimal invasiveness, and shorter recovery times compared to traditional surgical methods^12^. Despite these benefits, patients often experience post-procedure pain, requiring effective pain management strategies^13^. In most cases, the anesthetic choice for ablation of the liver is dependent on the surgical approach that is planned. More specifically, video-laparoscopic approaches require general anesthesia versus percutaneous approaches which can be performed with local infiltration and monitored anesthesia care (MAC). In general, video-laparoscopic approaches are typically indicated for the treatment of lesions that may be difficult to target via percutaneous puncture or the approach may be utilized when radio frequency ablation is performed as part of a staging procedure. In certain situations, general anesthesia may also be necessary for liver ablation. Recently, Beerman et al. reported their experience with ablation of liver lesions performed under general anesthesia^14^. Interestingly, the authors reported the use of high-frequency jet ventilation as a way of reducing the amplitude of respiratory movements to create near static conditions of both the upper-abdominal and intrathoracic organs allowing for greater surgical precision. Whenever possible though, the percutaneous treatment approach in combination with local infiltration and MAC is preferred. One anesthetic approach is to provide local anesthesia via infiltration at the puncture site and at the needle tract down to and including the glissonian capsule using 2% lidocaine^15^. Following infiltration of the local anesthetic, sedation is provided by administering one or more of the following: propofol, midazolam, diazepam, remifentanil or fentanyl^16^. Where available, a target-controlled infusion (TCI) regimen can be used to optimize the delivery of propofol and remifentanil and enhance recovery at the end of the procedure (doi: 10.1093/bjaed/mkv074). In situations where this is not available, the clinician can still provide adequate sedation using patient feedback and appropriate monitoring.

Butorphanol is a synthetic opioid analgesic that possesses both agonist and antagonist effects on opioid receptors^7^. It has been shown to effectively relieve moderate to severe pain without prominent respiratory depression^17^. Given its unique pharmacological profile, Butorphanol may be a suitable option for pain relief following microwave ablation, without compromising patient safety. First, this study selected a dosage of 4 mg Butorphanol for administration. One of the reasons is that patients reported minimal discomfort at this dosage in our preliminary trials. Our findings resonate with the study by Dinges et al.^18^, highlighting the significant analgesic potency of Butorphanol among short-acting opioid drugs. This underscores the pivotal role of Butorphanol in alleviating pain during and after procedures. The substantial reduction in the demand for additional analgesics both intraoperatively and postoperatively validates the early research by Chu^19^ and others regarding the efficacy of Butorphanol across various procedural settings. In our study, even those cases of percutaneous liver tumors, Butorphanol could alleviate the pain during the ablation of liver lesions.

Consistent with these findings, the notable divergence in intraoperative pain scores reaffirms the efficacy of Butorphanol. This difference remains conspicuously evident at the 6-h postoperative milestone, where the Butorphanol group exhibits significantly lower pain incidence compared to the control group. This aligns with the majority of prior studies^20,21^. The absence of significant adverse events related to Butorphanol aligns with the results of a previous meta-analysis by Zhihua Zhu and colleagues^22^, emphasizing its favorable safety profile—a crucial aspect of patient-centered interventions. It is suggested that in our study 4 mg of Butorphanol should be nasally instilled 5 min before surgery and additional 4 mg of Butorphanol could be available when the VAS was ≥ 4 during or after the surgery.

However, it's worth acknowledging the limitations of this study. While a fixed dosage of aids in control, there might be subtle differences in anesthetic effects among different individuals. Additionally, this may impact the reliability of exploring Butorphanol's adverse reactions such as nausea, vomiting, dizziness, and drowsiness.

In conclusion, this study confirms the potential of intranasally administered Butorphanol in pain management during and after microwave ablation procedures for liver cancer patients. Our findings beckon for further exploration, echoing the commitment to enhance our knowledge across multifaceted aspects of liver cell carcinoma treatment. Ongoing research, including extended follow-ups and a patient-centered perspective, holds the promise of unveiling the comprehensive impact of Butorphanol on postoperative comfort and patient well-being.

## Methods

### Study design and participants

This dual-center, randomized, placebo-controlled study was conducted at Shanghai Eastern Hepatobiliary Surgery Hospital and Nanjing Jinling Hospital from March 2023 to July 2023.

This study was approved by Ethics Committee of Eastern Hepatobiliary Surgery Hospital and Nanjing Jinling Hospital. All patients or their legal representatives provided written informed consent. This study was registered at Clinicaltrials.gov (Date of first registration: 11/09/2023, Registration number: NCT06031129). All methods were performed in accordance with the relevant guidelines.

Inclusion Criteria: patients performing Microwave Ablation sign the informed consent.

The exclusion criteria included patients with a body mass index > 30 kg/m2, a history of depression, opioid dependence, poorly controlled hypertension (systolic blood pressure > 180 mmHg), myocardial infarction, severe liver disease, and significant abdominal pain before surgery; patients with sensory system or language dysfunctions who could not cooperate to complete the scale; and pregnant women.

### Sample size calculation

We used PASS software to calculate the necessary sample size for this study. A cohort study reported that the pain score at 6 h after MWA treatment was 8.6 ± 2.7^6^. In our pretrial test, the score of visceral pain after MWA treatment at 6 h was 7.3 ± 1.2. Combining all the above data, we estimated that a 50% reduction of pain score in experiment group. Thus, 86 people were included in this trial (power = 80%, and α = 0.05). The ratio of the two sets of samples was 1:2. Considering an overall withdrawal rate of 10%, the sample size was estimated to be 300 patients (100 patients for control group and 200 patients for experiment group).

### Randomization

Patients were randomly assigned to receive normal saline (Control Group) or Butorphanol (Experiment Group) in a 1:2 ratio based on computer-generated stratified randomization numbers.

### MWA treatment

MWA treatment was performed by two experienced interventional radiologists having 5 and 26 years of experience in tumor ablation, respectively. Ultrasound (LOGIQ E9, GE Healthcare, Chalfont St. Giles, UK) guidance was used for tumor puncture with a 14G antenna (V4 applicator, Hospital services Spa, Aprilia, Italy). Tumors were treated following the manufacture’s protocol according to tumor size with ablation zone imaged through hyperechoic changes reaching the liver capsule at the end of the procedure. A single electrode was used for all treatments. Electrode was retrieved with tract ablation. Two days after the completion of the treatment, patients were discharged from the hospital. All patients had a CT scan at 1 month, and further follow-up was done in collaboration in between the interventional radiologist, and the medical oncologist.

### Perioperative management

The regimens were standardized in both groups. Patients were deprived of water for 2 h and fasted for 8 h before surgery. Patient’s intra- or post-operative visceral pain was evaluated with a 10 points pain visual analogic score (VAS), ranging from ‘0’ representing no pain to ‘10’ representing worst pain imaginable^23^. Patients in the Control group were given 5 mg of morphine intravenously 30 min before surgery, followed by 1 ml normal saline through nasal instillation 5 min before surgery. For patients in the Experimental group, 5 mg of morphine was also given intravenously 30 min before surgery and 4 mg of Butorphanol (1 ml) was nasally instilled 5 min before surgery. Additional painkiller, morphine for control group and Butorphanol for experimental group, was given during or after the surgery when the VAS was ≥ 4. If additional painkiller was needed during the procedure, a fixed dose at 5 mg of morphine or 4 mg of Butorphanol was given in each group.

The primary outcome was the incidence and extend of visceral pain during the ablation. And the secondary outcome was the pain level after surgery 6 h. Pain evaluation was defined according to the RESIST1.1. Complete relief (CR) was defined as the pain level after surgery 6 h was recovered to the level before surgery. Progressive disease (PD) was defined as the pain level after surgery 6 h was over 20% level during surgery. Partial relief (PR) was defined as the pain level after surgery 6 h was 30% lower than the pain level during surgery but not relief to the extend of preoperative pain level. Stable disease (SD) was defined as the pain level after surgery 6 h was between the standard of PD and PR.

### Statistical analysis

The data were analyzed using SPSS 26.0, and the figures were created by GraphPad Prism 9.0. All analyses were based on the intention-to-treat (ITT) principle. The Kolmogorov–Smirnov test was used to analyze continuous outcomes to judge the normality of their distributions.

Normally distributed continuous variables were summarized as the mean value ± standard deviation and were compared using independent t tests. Skewed continuous variables were summarized as the median value and interquartile range and were compared using the Mann–Whitney U test. As appropriate, categorical variables were summarized as the number and percentage and compared using the chi-square or Fisher’s exact test.

The primary endpoint was the incidence of visceral pain in the recovery room, and the chi-square test was used to compare the differences between the two groups. The risk ratios and 95% confidence intervals were reported for the primary and secondary outcomes. Mean arterial pressure (MAP), heart rate (HR), respiratory rate (RR) and pulse oxygen saturation (SpO_2_) were compared four times between the groups at T0, T1, T5, T10 and T15 using a two-sample Student’s t test.

Bonferroni correction was used to justify the P values for these three variables, and α level of 0.0125 was considered statistically significant. Moreover, α level of 0.05 was considered statistically significant for the remaining variables.

## Data Availability

Raw data, functional analysis sources and descriptions will be provided upon request. Correspondence should be addressed to GJ Qian. (E-mail: qianguojun1967@163.com.) and L Lu. (E-mail: mojingnanlulu@163.com), Tel&Fax: + 86-21-81870856, Address: Changhai Road 225, Shanghai, China.

## Abbreviations

CR: Complete relief
HCC: Hepatocellular carcinoma
HR: Heart rate
ITT: Intention-to-treat
MAP: Mean arterial pressure
MWA: Microwave ablation
PD: Progressive disease
PR: Partial relief
RFA: Radiofrequency ablation
RR: Respiratory rate
SD: Stable disease
SpO_2_: Pulse oxygen saturation
VAS: Visual analog scale
